# Cyclodextrin-Based Drug Delivery Systems for Depression: Improving Antidepressant Bioavailability and Targeted Central Nervous System Delivery

**DOI:** 10.3390/pharmaceutics17030355

**Published:** 2025-03-10

**Authors:** Renata Maria Văruț, Alin Iulian Silviu Popescu, Simina Gaman, Carmen Elena Niculescu, Adrian Ștefan Niculescu, Dalia Dop, Mioara Desdemona Stepan, Nina Ionovici, Cristina Elena Singer, Cristina Popescu

**Affiliations:** 1 Research Methodology Department, Faculty of Pharmacy, University of Medicine and Pharmacy of Craiova, 200349 Craiova, Romania; renata.varut@umfcv.ro; 2 Department of Internal Medicine, University of Medicine and Pharmacy of Craiova, 200349 Craiova, Romania; 3 Department I, Faculty of Dental Medicine, University of Medicine and Pharmacy of Craiova, 200349 Craiova, Romania; 4 Department of Mother and Baby, University of Medicine and Pharmacy of Craiova, 200349 Craiova, Romania; carmen.niculescu@umfcv.ro (C.E.N.); dalia.dop@umfcv.ro (D.D.); desdemona.stepan@umfcv.ro (M.D.S.); ninaionovici@yahoo.com (N.I.); cristina.singer@umfcv.ro (C.E.S.); 5 Department of Orthopedics, University of Medicine and Pharmacy Craiova, 200349 Craiova, Romania; niculescustefan94@gmail.com; 6 Department of Anatomy, University of Medicine and Pharmacy, Discipline of Anatomy, 200349 Craiova, Romania; cristina.popescu@umfcv.ro

**Keywords:** cyclodextrins, antidepressant drug delivery, bioavailability enhancement, pharmaceutical nanotechnology, targeted CNS delivery

## Abstract

Cyclodextrin (CD)-based drug delivery systems have emerged as a promising strategy to overcome limitations commonly encountered in antidepressant therapy, including low bioavailability, poor solubility, and suboptimal penetration of the blood–brain barrier. This review synthesizes current evidence demonstrating that complexing various classes of antidepressants—such as tricyclic antidepressants (TCAs), selective serotonin reuptake inhibitors (SSRIs), and atypical antidepressants—with β-CD or its derivatives significantly enhances drug solubility and stability. In addition, encapsulation with CDs can diminish systemic toxicity and improve pharmacokinetics, thereby helping to optimize dosage regimens and reduce adverse effects. Analysis of published in vitro and in vivo studies indicates that CD formulations not only boost therapeutic efficacy but also enable sustained or targeted release, which is critical for drugs requiring precise plasma and tissue concentrations. When compared to other carriers (e.g., liposomes, polymeric nanoparticles, dendrimers), CD-based systems often stand out for their ease of formulation, biocompatibility, and cost-effectiveness, although limited drug-loading capacity can be a drawback. We recommend expanding in vivo trials to substantiate the clinical benefits of CD–antidepressant complexes, particularly for treatment-resistant cases or specific subpopulations (e.g., elderly and pediatric patients). Additional investigations should also explore hybrid systems—combining CDs with advanced nano- or macroparticles—to amplify their advantages and address any limitations. Ultimately, integrating CDs into antidepressant regimens holds substantial potential to refine therapy outcomes, reduce adverse events, and pave the way for more personalized, effective interventions for depression.

## 1. Introduction

Depression is a serious mental illness affecting over 300 million people worldwide and contributing to approximately 800,000 suicides each year [1]. One of the leading causes of disability worldwide, Major Depressive Disorder (MDD), is marked by physical symptoms such as fatigue, weight loss, and appetite changes. A hallmark of MDD is anhedonia—the inability to feel pleasure—along with a lack of motivation, sleep disturbances, cognitive difficulties, and emotional symptoms like excessive guilt [2]. Depression stands among the most prevalent and burdensome mental health disorders worldwide, affecting individuals from diverse age groups and socioeconomic backgrounds.

Certain groups, including pregnant women, the elderly, and children, have a higher incidence of MDD, influenced by genetic, psychological, and social factors [3]. In some cases, depression is also linked to recurrent seizures, which can occur even during remission or persist beyond the duration of the disorder itself [4]. The clinical symptoms of MDD include a depressed mood, loss of interest, changes in weight or appetite, and an increased likelihood of committing suicide [5]. There is a certain correlation between the occurrence of MDD and social development and environment [6]. Considering these factors, it becomes evident how the COVID-19 pandemic may have significantly impacted the progression of depression in affected individuals, potentially exacerbating symptoms and influencing overall disease outcomes. Available data from the COVID-19 era can frighten us, with about one-fourth of COVID-19 patients globally commonly experiencing depression [7], and even more, one-half of the COVID-19 survivors suffer from depression [8]. Therefore, both COVID-19 and its mental effect (depression) require effective treatments concurrently. Antidepressants were prescribed very often before SARS-CoV-2 [9], and during the COVID-19 pandemic, usage increased [10]. However, some currently approved antidepressant drugs offer various side effects like restricted penetration, high toxicity, and low bioavailability [11,12], and medications are ineffective in one-third of patients with major depression [13]. While multiple therapeutic interventions exist, including psychotherapy and electroconvulsive therapy, pharmacological treatment remains the standard of care. Over the past several decades, various classes of antidepressant medications have been developed—most notably tricyclic antidepressants (TCAs), selective serotonin reuptake inhibitors (SSRIs), serotonin–norepinephrine reuptake inhibitors (SNRIs), monoamine oxidase inhibitors (MAOIs), and atypical antidepressants—each offering unique benefits as well as notable limitations. On the pharmacokinetic side, many antidepressants exhibit suboptimal water solubility, variable absorption, and extensive hepatic first-pass metabolism. These challenges translate into reduced and unpredictable bioavailability, often requiring higher or more frequent dosing. Additionally, for drugs that must cross the blood–brain barrier, limited permeability hampers therapeutic efficacy and necessitates dose escalations, which can elevate toxicity risks [14]. From a pharmacodynamic perspective, antidepressants can show delayed onset of action, which frustrates both patients and clinicians. Off-target receptor binding frequently gives rise to side effects that range from relatively mild (e.g., gastrointestinal discomfort) to more severe (e.g., anticholinergic effects, sedation, or cardiovascular complications). For instance, TCAs are well known for their anticholinergic burden, sedation, and narrow therapeutic index, creating a high likelihood of adverse events and potential toxicity at elevated doses. Conversely, SSRIs, while generally better tolerated, commonly induce sexual dysfunction, weight changes, and sleep disturbances. SNRIs can provoke increases in blood pressure, whereas MAOIs require stringent dietary restrictions to avoid hypertensive crises. Even atypical antidepressants, designed to sidestep some of these issues, can still exhibit sedation or weight gain, limiting patient adherence and long-term use [15]. These overlapping drawbacks underscore a pressing need for more efficient and safer methods to administer antidepressant drugs. Modern drug delivery platforms—including nanoparticle-based carriers, lipid vesicles, and CD-complex systems—are being investigated to alleviate the core challenges associated with conventional therapies. Enhancing solubility, improving bioavailability, and tailoring release profiles can facilitate more reliable drug concentrations at the central nervous system (CNS) target site, potentially minimizing peripheral side effects. By harnessing advanced delivery technologies, researchers aim to develop formulations that more effectively address depression’s therapeutic gaps, reduce systemic toxicity, and improve patient compliance [16].

Moreover, certain antidepressants are vulnerable to degradation by gastrointestinal enzymes, which may prevent them from reaching their target site intact, ultimately reducing their efficacy [17,18,19]. Within this context, CD-based carriers have garnered particular attention due to their biocompatibility and proven capacity to form stable inclusion complexes with poorly soluble active pharmaceutical ingredients. Recent investigations reveal that encapsulating antidepressant drugs within β-CD or its derivatives not only increases aqueous solubility but can also stabilize the drug, enable sustained or targeted release, and reduce systemic side effects. These findings have paved the way for a deeper examination of CD–antidepressant systems, which hold the potential to transform clinical practice by overcoming critical pharmacokinetic and pharmacodynamic hurdles [20,21]. Widely acknowledged within the scientific community, CDs are particularly valued for their ability to improve the solubility of poorly soluble drugs, making them a promising tool in pharmaceutical formulations aimed at overcoming drug delivery challenges [22,23,24]. The aim of this review is to explore the applications of CDs in the treatment of depressive disorders, examining their usage in combination with various antidepressant agents and assessing their potential to enhance CNS drug delivery. Furthermore, this paper aims to present and analyze the findings from relevant studies, providing insight into the effectiveness and therapeutic implications of CD-based drug delivery systems in depression treatment.

## 2. CDs

**Figure 1:**
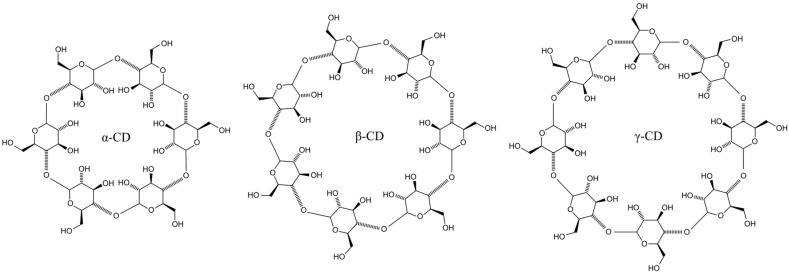
Chemical structures of CDs.

CDs, as illustrated in Figure 1, are water-soluble cyclic oligosaccharides that exhibit a truncated cone shape with a hydrophobic inner cavity and a hydrophilic exterior. They are composed of α-D-glucopyranose units linked by α-(1→4)-glycosidic bonds [25]. In nature, CDs are produced through the enzymatic degradation of starch by cyclodextrin glycosyltransferases, resulting in cyclic oligomers containing six (α-CD), seven (β-CD), or eight (γ-CD) glucose units [26,27,28]. The limited rotation of glycosidic bonds combined with the chair conformation of each glucose unit gives CDs their characteristic shape [29]. The inner surface, lined with skeletal C–H groups and ether oxygen atoms, contributes to its lipophilic properties, while the outer surface is rich in primary hydroxyl groups at C6 (forming the narrower edge) and secondary hydroxyl groups at C2 and C3 (forming the wider edge), thereby ensuring hydrophilicity. These amphiphilic characteristics enable CDs to encapsulate lipophilic drugs via the formation of host–guest inclusion complexes [30]. The stabilization of these complexes is achieved through conformational adjustments in the CD structure—such as steric relaxation of the ring—and a variety of non-covalent interactions, including van der Waals forces, hydrophobic interactions, dipole–dipole interactions, electrostatic forces, hydrogen bonding, and dispersion force [31,32]. Meanwhile, the hydrophilic outer surface of CDs enhances drug solubility by facilitating interactions with aqueous environments through hydroxyl groups. These properties make CDs highly suitable candidates for drug delivery systems [33].

Even with their capacity to improve solubility and bioavailability, CDs also present certain limitations. Due to their cavity size, CDs typically accommodate only a single drug molecule—either fully or partially—resulting in a relatively low drug-loading capacity [34,35]. However, the presence of highly reactive hydroxyl groups allows CDs to function as polyfunctional monomers, enabling their polymerization into cyclodextrin-based nano sponges [36]. Among these, β-CD is the most widely used due to its non-toxic nature, cost-effective production, and superior stability constants in drug complex formation [37,38]. The applications of cyclodextrin-based systems are highly versatile, extending across various fields and offering a wide range of potential uses in drug delivery and beyond. Recently, the formation of guest–host cyclodextrin complexes has been investigated for a diverse range of ligands, including remdesivir [39], water-soluble betulin derivatives [40], thiabendazole [41], ethinylestradiol [42], bis(1,10-phenanthroline) silver (I) salicylate [43], oncocalyxone A [44], and β-cyclodextrin-enhanced Eu^3+^ luminescent aggregates, which exhibit bright red fluorescence suitable for environmental detection systems [45], among many others [46,47,48,49]. Reactions involving cyclodextrins are still important to the separation [50,51] and the food industry [52]. Given the previously discussed challenges associated with antidepressant drugs, it is evident why numerous studies have focused on modifying their properties using CDs to enhance their stability, solubility, and overall therapeutic effectiveness. Lately, the interactions between antidepressant drugs and cyclodextrins have been examined [53,54,55], mostly to gain better water solubility of the drug [56,57,58] or to decrease its toxicity, or even for taste-masking [59].

### 2.1. Comparison of CDs with Other Drug Delivery Systems

**Figure 2:**
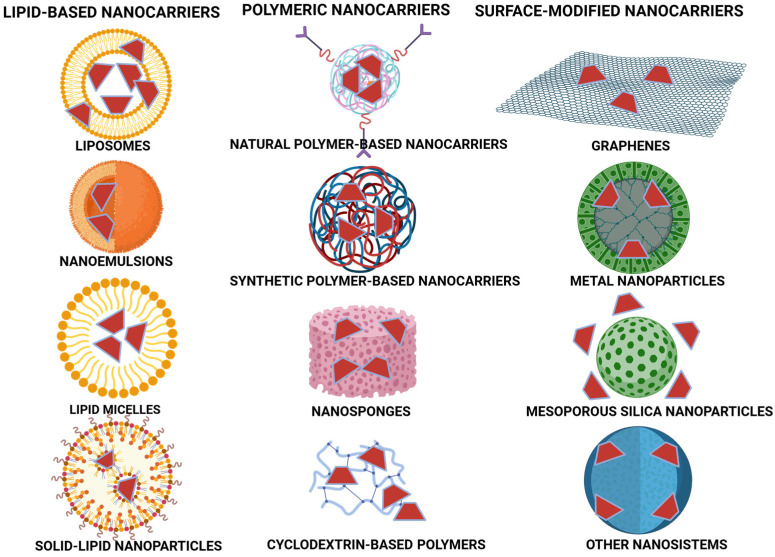
Classification of CD-modified nanomaterials for drug delivery, including CDs and their derivatives, drugs, polymers, and nanomaterials. Created with BioRender.com. https://app.biorender.com/illustrations/67c7148b33f12b7439b63714, accessed on 3 January 2025.

Significant advancements have been made in drug delivery methods over the last decade, with the introduction of carrier systems to improve how therapeutic agents work in the body over time and at effective levels. CDs are a type of molecule that enhances the solubility and stability of medications that struggle to dissolve in solution, and they are used in pharmaceutical studies due to their capacity to boost overall solubility, stability, and absorption rates, especially for drugs that are not easily soluble. Nevertheless, CDs are not nanocarriers employed for delivering drugs; conducting a comparison with alternative delivery methods, like liposomes, polymeric nanoparticles, and dendrimers, allows us to understand their strengths and weaknesses (Figure 2) [60].

Cyclodextrins primarily work by creating complexes with hydrophobic drugs to improve their solubility and shield them from enzymes that break them down in the body. This is especially helpful in developing antidepressants like fluoxetine and sertraline, along with antidepressants such as doxepin and clomipramine [47]. Nevertheless, TCAs, such as doxepin and clomipramine, can benefit from cyclodextrin-based formulations. However, cyclodextrins are subject to certain limitations, particularly their capacity to encapsulate only one drug molecule per complex. Furthermore, because they undergo rapid renal clearance, it may be necessary to modify cyclodextrin structures or combine them with other delivery systems to prolong their circulation in the body and ultimately improve their pharmacokinetic profiles [61]. In the realm of drug delivery systems, CNS liposomes are a popular choice worth mentioning. Phospholipid-based vesicles such as liposomes have the ability to carry both water and fat-soluble drugs effectively through the blood–brain barrier (BBB). Although extensive research has explored the potential of liposomes as nanocarriers for the delivery of serotonergic antidepressants, such as SSRIs and MAOIs, their widespread clinical application remains limited. The primary challenges associated with liposomal formulations include high production costs, physicochemical instability, and susceptibility to structural perturbations, which can lead to premature drug leakage and reduced shelf-life [62].

Polymeric nanoparticles have been extensively investigated for their potential in modulating drug release profiles. Nanoscale particles composed of biodegradable polymers, such as poly lactic-co-glycolic acid, can prolong systemic drug circulation and enhance bioavailability. These nanoparticles offer controlled and sustained drug release, which can improve therapeutic outcomes. However, their synthesis involves complex fabrication techniques, which may limit large-scale production. Additionally, concerns regarding cytotoxicity and long-term biocompatibility arise when non-biodegradable polymers are employed. Despite these challenges, polymeric nanoparticles hold promise in antidepressant formulations by optimizing drug delivery kinetics, enhancing CNS penetration, and reducing dosing frequency. Highly branched macromolecules known as dendrimers have emerged as promising nanocarriers for drug delivery due to their tunable surface chemistry and well-defined architecture. These nanoscale polymers offer high drug-loading capacity and can be functionalized with targeting ligands to enhance site-specific delivery, making them particularly suitable for CNS therapeutics. However, despite their advantages, concerns regarding cytotoxicity, immunogenicity, and high production costs have limited their clinical translation for antidepressant therapies. Currently, dendrimer-based formulations remain in the experimental phase, requiring further investigation to establish their safety, pharmacokinetics, and long-term therapeutic potential.

When comparing different drug delivery systems, each approach presents distinct advantages and limitations. CDs are widely recognized for their ease of formulation, cost-effectiveness, and ability to enhance solubility and bioavailability. However, their limited drug-loading capacity poses challenges for high-dose applications. On the other hand, liposomes and polymeric nanoparticles offer controlled release mechanisms and improved pharmacokinetic profiles, yet their complex manufacturing processes and stability issues remain significant hurdles.

Future advancements in antidepressant drug delivery may focus on hybrid approaches that combine CDs with liposomes or polymeric nanoparticles, integrating the benefits of multiple carrier systems while overcoming their respective limitations. A deeper understanding of these technologies is essential for optimizing antidepressant therapy, minimizing systemic side effects, and ultimately improving patient outcomes. As research progresses, CD-based nanotechnology formulations may pave the way for more targeted, efficient, and personalized treatments for depression and other CNS disorders [63,64].

### 2.2. Molecular Mechanisms of CD-Antidepressant Complexation

The reason that CDs can improve the solubility and stability of drugs lies in their structural design. This type of cyclic oligosaccharide features a hydrophobic space and a hydrophilic outer layer that enables it to create inclusion complexes with drugs through host–guest interactions. These interactions are mainly influenced by covalent forces like van der Waals forces, hydrogen bonding, and hydrophobic effects. They help keep the drug secure inside the CD cavity while also keeping it soluble in water.

Antidepressants, like TCAs and SSRIs, have shown promise in improving drug delivery efficiency through the use of CD-based formulations. Various studies have highlighted the impact of complexation between antidepressants and CDs in altering their properties. By improving drug solubility and stability, cyclodextrins help extend the drug’s half-life, reduce systemic toxicity, and facilitate its penetration into the central nervous system—factors that are all crucial for enhancing the efficacy of antidepressant therapy. A significant challenge in delivering drugs to the nervous system is overcoming the BBB. This specialized barrier restricts the passage of substances into the brain’s tissues and limits their effectiveness in treating neurological conditions such as depression. However, antidepressants combined with CD have shown an increased ability to pass through barriers, enabling drug delivery to the brain. Research on fluoxetine and paroxetine has shown that mixing them with HP-β-CD boosts their stability and the rate at which they dissolve. Research findings suggest that when dozepin is encapsulated within β-CD, it shows permeability and decreased degradation levels compared to its standalone form. This improvement is crucial in enhancing the drug’s effectiveness.

In addition to β-CD, there have been advancements in creating modified derivatives of CD with the aim of improving drug solubility and effectiveness for treatments. One such instance is the development and proven effectiveness of sulfobutylether-β-cyclodextrin (SBE-β-CD) in increasing nortriptyline’s solubility while preventing precipitation under bodily conditions. This careful adjustment in drug solubility and stability highlights the role played by selecting the CD derivative for each specific antidepressant medication.

The strategic planning of CD-based formulas offers a way to carefully manage the release rate of drugs in a controlled manner. This is especially beneficial for antidepressants with ranges; variations in drug levels in the blood could result in unwanted effects or decreased effectiveness. By adjusting the solubility and permeability of drugs using CDs, it is possible to have an idea of drug absorption patterns, reducing differences between individuals and improving treatment results [65,66].

### 2.3. Cyclodextrin-Based Controlled-Release Strategies in Antidepressant Therapy

Cyclodextrin-based formulations offer a versatile platform for creating controlled-release antidepressant delivery systems that can help maintain steady plasma levels of the drug and alleviate many of the side effects often associated with fluctuating drug concentrations [67,68]. By forming inclusion complexes with cyclodextrins—especially hydroxypropyl-β-cyclodextrin (HP-β-CD) or SBE-β-CD—antidepressants gain improved solubility, protection from premature degradation, and the potential for customized release kinetics [69,70]. For example, doxepin complexed with HP-β-CD has exhibited a sustained diffusion rate across biological membranes, translating into lower toxicity and enhanced analgesic efficacy—notably in neuropathic pain models, which demonstrates the broader application of this strategy for chronic conditions [71]. Similarly, duloxetine–SBE-β-cyclodextrin complexes have shown promise in transdermal delivery, increasing drug permeation and prolonging the therapeutic effect while minimizing systemic peaks and troughs [72]. These findings highlight how integrating CDs into antidepressant therapies may regulate the rate of absorption in a predictable manner.

Moreover, achieving consistent exposure is paramount for certain drug classes that are subject to variable absorption or extensive first-pass metabolism. In the realm of SSRIs and SNRIs, embedding agents such as fluoxetine, sertraline, or venlafaxine within cyclodextrin-based systems can mitigate issues like low aqueous solubility while also enabling extended-release properties [73,74]. Consequently, patients can benefit from reduced dosing frequency—a feature that may boost adherence—and maintain optimal CNS drug levels over a prolonged period. Adding to these advantages, combining cyclodextrins with nanoparticle carriers or hydrophilic polymer matrices can further refine drug diffusion profiles, thereby offering clinicians the option to tailor release kinetics to individual patient requirements. In practice, this could translate into better management of depression for individuals who are prone to noncompliance or who require tight control over plasma drug concentrations to avoid side effects such as sedation, hypertension, or sexual dysfunction [75].

Beyond SSRIs and SNRIs, other antidepressant classes also benefit from cyclodextrin-mediated delivery. Atypical agents and tetracyclic antidepressants, often limited by toxicity or low bioavailability, show improved pharmacokinetic and pharmacodynamic profiles upon complexation with β-CD or its derivatives [76]. For instance, multi-equilibrium systems containing sertraline and β-cyclodextrin have demonstrated enhanced solubility alongside a more predictable release curve in aqueous solution. Additionally, orodispersible tablets of fluoxetine that use cyclodextrins for taste masking and modulated dissolution illustrate the translational viability of these approaches in actual pharmaceutical product development. Ultimately, such strategies address key challenges in antidepressant therapy, particularly interpatient variability in absorption, gastrointestinal degradation, and the need for sustained CNS targeting [77].

Recent investigations have also illustrated how SBE-β-CD can improve the controlled-release profiles of TCAs, including nortriptyline and imipramine, leading to more uniform plasma concentrations and fewer side effects stemming from excessive peak levels [78]. In this context, the choice of cyclodextrin derivative—whether SBE-β-CD, methyl-β-cyclodextrin, or HP-β-CD—plays a pivotal role in fine-tuning the drug’s release kinetics. Adapting the CD cavity size, degree of substitution, or surface modification enables precise control over how an active ingredient is encapsulated, released, and ultimately distributed throughout the body. This versatility opens avenues for the future development of hybrid technologies that merge cyclodextrins with lipophilic nanoparticle cores or biodegradable polymers capable of promoting controlled release, selective tissue targeting, and reduced off-target toxicity. When applied to atypical antidepressants (e.g., lurasidone, mirtazapine), these hybrid systems could target specific brain regions, further enhancing efficacy while minimizing peripheral effects such as metabolic changes or cardiovascular risks [79].

Researchers have leveraged this approach for oral, intranasal, and other administration routes, noting that certain CD complexes can bypass extensive hepatic metabolism, accelerate drug onset, and promote higher concentrations of antidepressants in the brain. The intranasal delivery of CD-based formulations is especially noteworthy for its potential to enable direct nose-to-brain transport, minimizing gastrointestinal degradation and bypassing the blood–brain barrier. Studies involving herbal-derived antidepressant compounds such as hypericin, complexed with β-cyclodextrin, demonstrate how intranasal delivery can accelerate drug action while decreasing the systemic dose required, thereby lowering the probability of unwanted effects. Another noteworthy example involves intranasal berberine–HPβCD complexes, which showed higher intracerebral bioavailability than conventional administration forms, with marked enhancements in monoamine neurotransmitter levels in the hippocampus [80].

Notably, several recent preclinical investigations have expanded upon the intranasal concept by evaluating SSRIs such as paroxetine or citalopram in CD-based nasal sprays or gels. These studies documented faster onset of antidepressant-like effects in rodent models, coupled with reduced peripheral drug exposure—an outcome that holds promise for patients who require rapid symptom relief while avoiding dose-dependent side effects [81]. In another example, intranasal venlafaxine loaded into a β-CD functionalized nanoparticle demonstrated superior hippocampal targeting and greater inhibition of depressive-like behaviors compared to oral venlafaxine, supporting the potential for clinical translation. Such intranasal formulations can be especially beneficial in cases where oral administration is compromised by gastrointestinal issues or for individuals who may have difficulty adhering to standard oral regimens [82].

Hybrid cyclodextrin–polymer complexes, sometimes referred to as cyclodextrin-based nanosponges, are another strategy garnering attention. These nanosponges are cross-linked polymeric networks built from cyclodextrins, thus offering multiple cavities and nanochannels for drug encapsulation [83]. Although widely investigated for applications beyond CNS disorders, early studies suggest that these systems can release certain antidepressants over days rather than hours, potentially stabilizing patients’ moods more effectively. Dendrimer-based approaches may yield even more sophisticated carriers by conjugating cyclodextrins to branched polymer architectures. This design enables multivalent drug loading and, with suitable surface modifications, active targeting of sites of interest, such as specific regions of the brain. Although dendrimers can sometimes pose risks owing to their synthetic complexity and potential cytotoxicity, researchers have shown that decorating the surface with polyethylene glycol or other functional groups can enhance biocompatibility [84].

Taken together, these findings substantiate the view that CD-based, controlled-release platforms represent a pioneering approach to improving depression therapy by increasing efficacy, reducing adverse events, and enhancing patient compliance. Overall, the integration of cyclodextrins into controlled-release formulations addresses several major limitations that have long hindered antidepressant therapy, including suboptimal solubility, variable absorption, and difficulties in achieving sustained CNS drug levels. While many proposed systems remain in preclinical or early-stage clinical research, the pathway to practical adoption involves a thorough evaluation of safety, pharmacodynamics, and real-world efficacy. Nevertheless, the advancements in cyclodextrin-based complexes and hybrid nanoparticle approaches are rapidly converging toward novel depression treatments that are both more potent and better tolerated than current standards of care. This evolving evidence base sets the stage for next-generation antidepressant therapies that leverage nanotechnology and, specifically, cyclodextrins to deliver targeted, consistent, and personalized treatment for individuals with major depressive disorders [85].

## 3. Factors Affecting Depression

Depression ranks among the most prevalent mental illnesses worldwide and is a principal cause of disability [86]. It develops through an interplay of sociological, psychological, and biological risk factors, ranging from early-life trauma and chronic stress to inflammation-driven changes in brain function [87]. Inflammation can elevate proinflammatory hormones and cytokines, triggering oxidative stress that compromises cognitive and executive processes. Biomarkers, in turn, have proven critical for assessing both the severity and progression of depression.

Early and repeated exposure to adverse events—particularly trauma or abuse—can reconfigure how the brain interprets stress and emotions, thereby intensifying susceptibility to depression [88]. In addition, social isolation, loneliness, and inadequate sunlight have been identified as significant predictors of depressive episodes, with some investigations reporting that around 2.4% of the variance in depressive symptoms is linked to isolation, while loneliness accounts for nearly 9.7%. Insufficient natural light, especially in winter, is also correlated with seasonal affective disorder [89,90]. Socioeconomic standing further influences depression risk: constraints in resources and opportunities, as well as heightened vulnerability to life stressors, exacerbate depressive outcomes [91]. Interestingly, higher-income groups likewise face their own risk factors, demonstrating that depression does not discriminate based on economic status [92,93]. Chronic stress can disrupt the hypothalamic–pituitary–adrenal axis and contribute to structural changes in the hippocampus—an area intimately tied to emotion regulation—thus perpetuating a vicious cycle of worsening mental and physical health [94,95,96].

During the COVID-19 pandemic, depression became even more conspicuous. Approximately 12.8% of recovered patients experienced depressive symptoms traceable to both the virus and pandemic-related stress [97,98,99]. Specific indicators—such as female sex, existing psychiatric histories, and degree of inflammatory response—magnify risk, explaining the 11–28% prevalence of depression symptoms reported in survivors [100,101]. Such data reveal the intricate relationship between virological factors, stress, and mental well-being.

Given depression’s substantial socioeconomic toll, including an annual productivity loss exceeding one trillion dollars [102], expanding access to effective interventions is essential. Yet financial and societal barriers hinder early intervention, as low- and middle-income nations spend below 1% of total health budgets on mental health, leaving over half of those affected without adequate care [92]. Strengthening early detection, reducing stigma, and investing in treatment may mitigate both human suffering and broader economic repercussions. The main antidepressants are SSRIs, SNRIs, TCAs, MAOIs, atypical antidepressants, NMDA receptor antagonists, and serotonin modulators, each offering distinctive pharmacological mechanisms and clinical benefits (Table 1).

## 4. Overview of Different Antidepressant Drugs Combined with CDs

### 4.1. TCAs

Amoxapine (AXP): A study found that the aqueous solubility of AXP improves due to forming a complex with ɣ-CD. As a result, the inclusion complex of AXP-ɣ-CD shows great promise for developing an innovative formulation of AXP for drug delivery. This approach has the potential to expand the clinical applications of AXP in the pharmaceutical industry and biomedical sciences [103].

Clomipramine (CPM): A study reported the encapsulation of clomipramine with β-CD, highlighting the role of intermolecular C/O–H interactions in stabilizing the β-CD–CPM complex. Thermodynamic stability, evaluated through density functional theory (DFT) calculations, suggests its potential use in drug delivery systems [57,66].

Doxepin (DXP): Similar findings were reported for doxepin, where β-CD complexation contributed to its stabilization through intermolecular interactions. Computational analysis confirmed that these inclusion complexes could enhance drug formulation and delivery. Another comparative research study on the transcutaneous delivery of doxepin using a pure drug solution (PDS) and a doxepin–HP-β-CD complex solution (CDS) in a porcine epidermis model found that electroporation enhanced drug retention in both cases. However, while PDS led to rapid release, CDS provided sustained release, resulting in prolonged analgesic activity in rats. These findings highlight the potential of CDS for noninvasive, sustained drug delivery in chronic pain conditions like postherpetic neuralgia [54].

Desipramine (DPM): A study investigated the complexation of desipramine with β-CD, revealing that the A and C rings are embedded in the β-CD cavity, with the A-ring positioned near the O6 side at an almost right angle to the O4 plane. The complex is stabilized by C–H···π interactions and N–H···O hydrogen bonds, contributing to its structural stability and potential for enhanced therapeutic effects [104].

Imipramine (IPM): Similar findings were observed for imipramine as for deimipramine, where β-CD encapsulation followed a comparable inclusion pattern. Computational and spectroscopic studies suggested a bimodal complexation mechanism, enhancing the drug’s stability. These results align with previous findings on nortriptyline and amitriptyline [73], reinforcing the role of β-CD in stabilizing TCAs and improving their drug delivery potential.

Nortriptyline (NRT): Permeability and partition were studied using two types of β-CD. The investigation of nortriptyline hydrochloride (NTT•HCl) in the presence of cyclodextrins revealed a reduction in drug transfer from the aqueous to organic phases. The effect was more pronounced with SBE-β-CD than with 2-HP-β-CD, highlighting differences in interaction strength. Additionally, the use of the lipophilic PermeaPad barrier provided a more comprehensive assessment by simulating interactions not only with cyclodextrins but also with components of biological membranes, offering deeper insights into the drug’s permeability profile [105].

Protriptyline (PTR): A study that presents the first crystallographic confirmation of protriptyline (PRT) stabilization within the β-CD cavity was found. The findings highlight the potential of β-CD to enhance the bioavailability and therapeutic application of TCAs, suggesting its promising role in drug delivery systems [106].

In Table 2, we showcase multiple TCAs complexed with β-cyclodextrin (β-CD) or other cyclodextrin derivatives. Collectively, these studies primarily examine how variations in drug-to-CD molar ratios (e.g., 1:1, 2:1) and experimental conditions (pH, temperature, and solvent systems) impact both the stability and solubility of the resulting inclusion complexes. For example, work on DPM reveals that a 1:1 ratio with β-CD can significantly enhance aqueous solubility, whereas a 2:1 ratio potentially improves complex stability yet may alter release profiles [104,107,108,109,110]). Similarly, IPM shows greater dissolution and chemical stability when encapsulated in β-CD at a 1:1 ratio, underscoring the importance of stoichiometry (references [104,109]). NRT, studied in both α-CD and β-CD, demonstrates improved bioavailability indicators—such as reduced tendency to partition into organic solvents—although these benefits can differ based on which cyclodextrin derivative is used. Most investigations support the premise that cyclodextrin encapsulation enhances solubility, modifies drug release kinetics, and may reduce cytotoxic side effects by limiting free drug concentration. Nevertheless, these studies share several limitations. First, the majority are in vitro assessments, which cannot fully capture the complexity of human absorption and metabolism. Second, variations in experimental design (different media compositions or temperature ranges) make direct comparisons challenging. Third, few studies include comprehensive in vivo validation, leaving open questions about pharmacokinetics, pharmacodynamics, and actual clinical efficacy. Finally, most published work focuses on short-term endpoints, providing limited insight into how these complexes behave during prolonged administration—especially relevant given that many TCAs require long-term usage.

Despite these constraints, the collective findings suggest that TCA–CD inclusion complexes could address hurdles such as suboptimal solubility or limited bioavailability. Future research should concentrate on rigorous in vivo trials with standardized methodologies to confirm clinical benefits and optimize formulations. Moreover, exploring additional cyclodextrin derivatives and hybrid nanosystems may further improve targeted delivery and release control, thereby streamlining TCA-based therapies [111,112,113,114].

### 4.2. SSRIs

Fluoxetine (FLX): Unlike other studies that utilized CDs for drug delivery, one study employed CD-modified capillary electrophoresis for the chiral separation of fluoxetine. Various native and derivatized CD derivatives, both neutral and ionized, were screened to identify the optimal chiral selector, successfully demonstrating the effectiveness of this approach [115]. Many studies on where CDs were used for drug delivery were also found [116].

Sertraline (SRT): In this case, studies have explored both the thermodynamic properties and drug delivery potential of sertraline–CD complexes [117]. DFT calculations suggest that CD inclusion enhances the thermodynamic stability of sertraline, with the drug’s conformational flexibility playing a role in its pharmacological function. Structural comparisons between the free salt form, the CD complex, and protein-bound states further support these findings [118]. Other studies used CDs for the chiral separation of sertraline by microemulsion electrokinetic chromatography or for increasing the solubility and found that SRT in the presence of CDs at 1:1 and 1:2 molar ratios was more soluble than free SRT [119].

Paroxetine (PXT): A study comprehensively investigated the conformational flexibility and stability of PXT for the first time, revealing its true nature as an SSRI. The findings indicated that the PXT: β-CD complex in a 1:1 ratio was less stable than the 2:1 ratio [120].

### 4.3. Atypical Antidepressants

Mianserin (MIA): Studies have explored the stability and biological effects of mianserin–CD complexes using different methods. DFT calculations assessed the thermodynamic stability of the complex, while another study employed isothermal titration calorimetry and cytotoxicity testing on Chinese hamster B14 cells. The results showed that cell viability remained above 80% when cultured with dimethyl-β-cyclodextrin (DM-β-CD) alone but decreased when mianserin was introduced. Interestingly, the presence of DM-β-CD increased MIA’s toxicity, contrasting with the protective effect observed in the β-CD complex [55].

Mirtazapine: In a notable study by Wen and colleagues, the authors employed cyclodextrin-based enantioselective separation for mirtazapine and its metabolites by means of capillary electrophoresis with acetonitrile field-amplified sample stacking. This approach harnessed the unique host–guest interactions of cyclodextrins, which selectively bind to the enantiomers and thus improve resolution during separation. By optimizing experimental parameters such as pH and buffer composition, the researchers achieved efficient chiral discrimination and reliable quantification of the drug’s enantiomers. Such work not only highlights the valuable contribution of cyclodextrins to advancing analytical methodologies for complex antidepressants but also underscores the broader significance of enantioselective analysis in refining pharmacokinetic and pharmacodynamic studies [121].

Trazodone: A study explored the use of β-CD and γ-CD as electroactive materials in a novel sensing system for trazodone. This was the first investigation of its kind, demonstrating that the developed method was more sensitive, accurate, reproducible, and robust compared to previously proposed ion-associated techniques [122].

### 4.4. Other Antidepressant Drugs

Sulpiride: The study investigated its electrochemical behavior using a pyrolytic graphite electrode modified with graphene oxide and β-CD. The developed nanosensor showed a significant increase in the signal of sulpiride compared to the bare electrode. The method was fast, simple, selective, and inexpensive and offered shorter analysis times compared to other published methods [53].

Maprotiline (MPL): We found that many studies provide the first crystallographic evidence of MPL stabilized within the β-CD cavity. These findings suggest that β-CD can enhance the bioavailability and therapeutic application of tetracyclic antidepressants, highlighting its potential for efficient drug delivery [105].

### 4.5. Agents with Antidepressant Proprieties

Neoponcirin (NEO): Neoponcirin is the 2S-5-hydroxy-4′methoxyflavanone-7-O-β-glucopyranosyl-(1→6)-β-rhamnoside. It is one of the main constituents of *Clinopodium mexicanum* [123]. As with many flavonoid glycosides, NEO is effective when intraperitoneally or intraventricularly administered, but oral treatment with NEO is ineffective in inducing anxiolytic effects, limiting its use in humans [124,125]. These limitations were explored by a study where it was found that the NEO/β-CD treatment did not result in notable alterations in the growth of mice. These findings suggest that the complex alleviated the effects of stressors, reducing their impact on the physiological and behavioral responses of the experimental subjects. Nevertheless, no obvious signs of toxicity were detected; the animals tolerated the treatments and were able to overcome the behavioral challenges. Acute oral administration with NEO/β-CD caused antidepressant- and anxiolytic-like effects in mice. The repeated oral administration of NEO/β-CD produced positive benefits in mice under prolonged stress, causing anxiolytic and antidepressant-like effects [126]. This suggests that this approach could be a promising avenue for future treatments.

*Nigella sativa* (NS): NS, or black seed, is considered a potent antidepressant due to its rich source of compounds called thymoquinone [127,128]. Thymoquinone has been reported to have antioxidant and anti-inflammatory properties, influence neurotransmitters, alter mood regulatory pathways, and improve depressive symptoms [129,130]. Among the studies conducted, some have investigated the antidepressant potential of a lyophilized nanosuspension of *Nigella sativa* (NSOR) oleoresin complexed with HP-β-CD. The in vitro drug release studies from nano-NSOR were performed for up to 72 h. The results showed a significant release of thymoquinone (90.15%) from nano-NSOR [131].

### 4.6. Clinical Implications and Future Perspectives

In addition, by improving both the efficacy of medications and their absorption in the body, cyclodextrin-based formulations are opening new directions for innovative approaches to treating depression. Depression is a condition characterized by varying responses to drug treatment among patients. Genetic variations in enzymes for drug metabolism, like cytochromes known as CYP enzymes, can impact how antidepressants are processed in the body, leading to either reduced effectiveness or an increased potential for side effects. CD-complexed formulations could potentially provide a solution by offering a foreseeable pharmacokinetic profile that helps reduce the uncertainties linked to genetic variations in drug metabolism [132].

Pharmacogenetic research indicates that individuals identified as poor metabolizers or ultrarapid metabolizers when taking antidepressants may face challenges in achieving effective treatment results. Poor metabolizers may encounter increased side effects due to drug buildup in their system, while ultrarapid metabolizers might experience reduced drug effectiveness due to clearance rates. Antidepressant formulations based on controlled release could assist in regulating drug absorption and availability in the body to ensure plasma levels regardless of variations. For instance, to illustrate the point further, containing medications such as fluoxetine or venlafaxine within HP-β-CD has been demonstrated to extend the release of the drug and reduce fluctuations that lead to side effects or a decrease in effectiveness [133].

Moreover, medications combined with cyclodextrins exhibit stability against enzymes, potentially decreasing the requirement for altering doses in individuals with variations affecting drug metabolism. This feature makes formulations based on cyclodextrins highly attractive for tailored antidepressant treatment guided by genetics, where customized therapeutic plans are essential for enhancing treatment results. Apart from factors influencing drug response, research is actively exploring cyclodextrin formulations enhanced by nanotechnology for their capacity to specifically target the brain and spinal cord. Traditional antidepressants often face challenges in achieving effective brain penetration due to their limited lipophilicity and susceptibility to active efflux mechanisms, such as P-glycoprotein, at the BBB. Scientists are looking into modified forms of CD-altered versions of beta cyclodextrin derivatives because they may help drugs get past or block these removal mechanisms in order to deliver medications effectively to the CNS. For example, β cyclodextrin nanoparticles modified with blood–brain barrier-targeted molecules, like transferrin or glutathione, have been studied for facilitating the transportation of antidepressants across the blood–brain barrier actively. This approach shows potential in decreasing the systemic dosages needed to reach therapeutic levels in the brain effectively. Regarding regulations, the use of CD-based drug formulations is not a recent development. Many drug combinations involving CDs have received approval from the FDA and EMA in fields like oncology and antifungal medications (such as amphotericin B with CDs and paclitaxel with CDs). However, the use of CDs in treating depression is still in an early phase. They need thorough clinical trials to confirm their safety and effectiveness compared to conventional treatments in the long run [134].

One of the areas of exploration in this field involves creating dual drug–CD complexes that combine two complementary antidepressants or a mix of antidepressant and antipsychotic drugs within a single CD structure. This innovative method could boost the effects between the drugs while lessening interactions between them and making it easier for patients to stick to their medication schedules by simplifying dosage routines. It is crucial to move beyond laboratory and animal models and evaluate these cyclodextrin-formulated antidepressants in humans. Rigorous in vivo studies (in relevant animal models of depression) should precede well-designed clinical trials to confirm that the improvements in solubility, bioavailability, and efficacy indeed manifest in human physiology [135].

Large-scale clinical trials will need to compare cyclodextrin-based formulations with standard antidepressant treatments to verify superior outcomes or reduced side effects in patients. Only through such trials can the long-term safety and therapeutic advantages be conclusively established, which is a prerequisite for clinical adoption. Engaging with regulatory authorities early in the development process will smooth the path toward approval. Notably, certain cyclodextrin-enabled formulations (for example, cyclodextrin complexes used in antifungal and oncology drugs) have already earned FDA and EMA approvals. Leveraging this precedent, researchers and pharmaceutical developers should prepare comprehensive data on the safety, quality, and efficacy of antidepressant–cyclodextrin complexes. This includes chronic toxicity studies and assessments of any cyclodextrin-related excipient effects, given that long-term use is expected in depression management. Regulatory considerations will also entail demonstrating that the manufacturing process can consistently produce a stable, high-quality product. Overall, providing robust evidence that cyclodextrin-based delivery is not only effective but also meets all safety standards will be key to obtaining regulatory approval for clinical use [136].

Furthermore, customized compact discs containing BBB-focused connectors signify a period in treating mental health disorders. This enables the distribution of medication to the nervous system while reducing overall toxicity. With studies, these advancements have the potential to revolutionize how antidepressants are developed and used, providing individuals with safer, more efficient, and tailored treatment choices [137,138,139].

## 5. Conclusions

In summary, the studies reviewed in this work collectively demonstrate that cyclodextrin-based drug delivery systems have significant potential to overcome key challenges in antidepressant therapy, including poor aqueous solubility, limited bioavailability, and inadequate CNS penetration. The ability of cyclodextrins—especially β-cyclodextrin and its derivatives—to form stable inclusion complexes with various antidepressants has been shown to enhance drug solubility, chemical stability, and controlled release profiles. These preclinical investigations, both in vitro and in vivo, underscore the promise of cyclodextrin-based formulations in optimizing pharmacokinetic and pharmacodynamic outcomes, thereby potentially reducing systemic toxicity and improving therapeutic efficacy. Despite these promising findings, several limitations remain. Many of the studies are constrained by variability in experimental conditions and rely predominantly on in vitro models that do not fully replicate the complexity of human physiology. As such, there is a pressing need for standardized methodologies and comprehensive in vivo investigations. Future research should focus on advancing these formulations into well-designed clinical trials to validate their efficacy and safety in real-world settings. In addition, exploring hybrid drug delivery systems that combine cyclodextrins with other nanotechnologies may further enhance drug targeting and release kinetics. Integrating pharmacogenetic profiling into the design of these systems could also facilitate personalized treatment approaches, ultimately paving the way for more effective and tailored interventions for depression and other CNS disorders.

## Figures and Tables

**Table 1:** The classification of antidepressant drugs.

Class	Drug
SSRIs	Fluvoxamine, Paroxetine, Escitalopram, Citalopram, Sertraline, Fluoxetine
SNRIs	Venlafaxine, Desvenlafaxine, Duloxetine, Levomilnacipran, Milnacipran
TCAs	Imipramine, Nortriptyline, Amitriptyline, Clomipramine, Desimipramine, Doxepin, Amoxapine, Protryptiline
MAOIs	Phenelzine, Tranylcypromine, Isocarboxazid, Selegiline
Atypical Antidepressants	Bupropion, Mirtazapine, Trazodone, Nefazodone, Vortioxetine, Vilazodone, Mianserine
NMDA Receptor Antagonists	Esketamine, Ketamine
Serotonin Modulators	Trazodone, Nefazodone, Vortioxetine, Vilazodone

**Table 2:** Summary of CD-TCA complex studies.

Study (Year)	Drug (Guest)	Cyclodextrin Type	Study Findings	Study Limitations
2020 [107]	Desipramine	β-CD (1:1)	X-ray crystallography showed the drug’s aromatic rings fit into the β-CD cavity, stabilized by C–H···π and N–H···O interactions, indicating a stable 1:1 complex and suggesting enhanced aqueous solubility	Structural analysis only; no in vivo data to confirm improved bioavailability (study was purely in vitro)
2020 [104]	Imipramine	β-CD (1:1)	β-CD encapsulation yielded a similar inclusion complex as with desipramine, improving drug stability. The complex formation reinforces β-CD’s role in stabilizing TCAs and potentially enhancing delivery	In vitro characterization without in vivo validation; primarily a structural study without direct pharmacokinetic data
2015 [108]	Desipramine	β-CD (1:1)	Confirmed formation of a 1:1 inclusion complex that significantly increased desipramine’s aqueous solubility and was explored for improved pharmaceutical formulation	Experiments were laboratory-based; lacked any in vivo study to demonstrate actual improvement in drug absorption or efficacy
2014 [109]	Desipramine	β-CD (2:1)	Spectrofluorimetric analysis showed enhanced fluorescence of desipramine with β-CD, indicating complex formation. A 2:1 drug:CD ratio improved complex stability but altered the drug release profile.	Analytical study focusing on detection; did not evaluate therapeutic effects or long-term stability in biological systems (no in vivo component)
2016 [110]	Desipramine	β-CD (1:1)	Spectroscopic characterization confirmed 1:1 inclusion complexation of desipramine with β-CD, supporting improved stability of the drug in solution	Findings are based on solution-phase spectroscopic data; no evaluation of how the complex behaves in vivo or in a full dosage form
2001 [104]	Desipramine	β-CD (1:1)	Observed that desipramine tends to self-aggregate in aqueous solution, and β-CD inclusion disrupts these aggregates by forming stable 1:1 complexes	Study in aqueous solution only; did not address drug behavior in vivo or in complex biological fluids (focus was on physico-chemical interactions)
2020 [111]	Nortriptyline	β-CD (1:1)	Single-crystal XRD and DFT demonstrated a stable 1:1 β-CD–nortriptyline complex. Inclusion reduced the drug conformational flexibility (“butterfly” angle) and improved its stability, supporting the idea that CD encapsulation could reduce nortriptyline’s side effects	Structural and theoretical study only; no direct measurement of pharmacological outcomes or drug release in vivo was performed
1993 [112]	Nortriptyline	β-CD (1:1)	Using a β-CD-bonded HPLC column, this study achieved separation of nortriptyline, indicating formation of an inclusion complex during chromatography. It provided early evidence of specific nortriptyline–β-CD interactions in solution.	Focused on chromatographic behavior rather than therapeutic application; results are method-specific and not translated to actual drug delivery or bioavailability improvements
1993 [109]	Maprotiline	β-CD (1:1)	Reported that maprotiline can form a 1:1 complex with β-CD, similarly to other TCAs, which is expected to enhance its water solubility and stability in solution (by analogy with other drugs in the study)	The investigation was limited in scope (in vitro); no direct data on how the complex affects maprotiline’s pharmacokinetics or efficacy in vivo in vivo was provided
2017 [113]	Nortriptyline	β-CD (1:1)	Prepared and characterized a nortriptyline–β-CD inclusion complex. The authors noted enhanced solubility and suggested that complexation may mitigate dose-related side effects and improve patient compliance by more controlled drug release.	Results are based on in vitro analyses; the study did not include in vivo tests to confirm reduced side effects or improved therapeutic outcomes in practice
1992 [114]	Nortriptyline	β-CD (1:1)	Ion-selective electrode studies quantified the binding of nortriptyline with β-CD, confirming 1:1 complex formation. The presence of β-CD reduced nortriptyline’s tendency to partition into a non-aqueous phase, indicating improved aqueous retention.	Entirely in vitro measurement of binding affinity; did not examine the complex in biological systems or assess actual improvements in drug absorption
1992 [104]	Nortriptyline	α-CD (1:1)	Showed that nortriptyline also forms a 1:1 inclusion complex with α-cyclodextrin, though the smaller α-CD cavity may lead to a different binding affinity. Complexation efficacy differed by cyclodextrin type, underscoring the influence of CD size on stability.	In vitro chemical study; the relative benefit of using α-CD over β-CD was not confirmed in a biological context, and the smaller CD efficacy remains theoretical without in vivo data
1992 [104]	Maprotiline	β-CD (1:1)	Found that maprotiline forms a stable 1:1 complex with β-CD, which likely increases its solubility in water. This result is in line with other TCAs forming inclusion complexes with β-CD, hinting at improved delivery potential for maprotiline as well.	Based on in vitro binding studies; no follow-up in vivo research was conducted to verify any improvement in maprotiline’s pharmacological profile or reduction in side effects
1992 [104]	Maprotiline	α-CD (1:1)	Demonstrated that maprotiline can also complex with α-CD in a 1:1 ratio, although the smaller cavity may not accommodate the drug as effectively as β-CD. This suggests that cyclodextrin cavity size is critical for optimal inclusion.	Only investigated under laboratory conditions; it remains unclear how well the α-CD complex of maprotiline would perform in vivo or if it offers any therapeutic advantage over the β-CD complex
1992 [104]	Protriptyline	β-CD (1:1)	Early evidence	Initial findings were preliminary and in vitro; the study did not provide in vivo data, and robust structural confirmation came only in subsequent research years later

## Data Availability

Data are contained within the article.
